# A mediation analysis of family members’ knowledge, attitudes, and practices in nutritional and dietary management for gastric cancer patients

**DOI:** 10.3389/fmed.2025.1680862

**Published:** 2026-01-09

**Authors:** Cailian Liu, Min Wei, Lijuan Song

**Affiliations:** Department of Gastroenterology, Inner Mongolia Hospital, Peking University Tumor Hospital, Hohhot, China

**Keywords:** knowledge, attitude, practice, family member, nutritional care, gastric cancer

## Abstract

**Background:**

Gastric cancer is a leading cause of global cancer mortality, especially in China, with high incidence and death rates. Nutritional status is crucial for prognosis, yet adherence to dietary guidelines remains low. Family caregivers, pivotal in patient care, often lack adequate nutritional knowledge and practices, highlighting the need for targeted interventions to improve outcomes. This study aimed to investigate the knowledge, attitudes, and practices (KAP) of family members regarding nutritional care for gastric cancer patients.

**Methods:**

A cross-sectional study was conducted between January and May 2024 at the Department of Medical Oncology, Inner Mongolia Hospital, Peking University Cancer Hospital. Demographic information and KAP scores were collected using structured questionnaires.

**Results:**

Among 406 valid questionnaires, 216 (53.2%) respondents were female. Median (25, 75%) knowledge, attitude, and practice scores were 27.00 (19.00, 33.00), 31.00 (30.00, 36.00), and 36.00 (34.00, 42.00), respectively. Structural equation modeling revealed that factors like income, nursing support, and training significantly influenced knowledge, attitude, and practice (all *p* < 0.05). Mediation analyses showed indirect effects of these factors on attitude and practice (all *p* < 0.05).

**Conclusion:**

Family members understand gastric cancer nutritional care and maintain positive attitudes, but their practices are inadequate. Educational interventions are needed to improve caregiver knowledge and implementation.

## Introduction

Gastric cancer represents a significant global health burden. In 2015, it was the fifth most common cancer globally, with 1,033,701 new cases and 782,685 deaths, making it the third leading cause of cancer death worldwide (1). In China, gastric cancer is particularly prevalent, ranking as the second most common cancer. In 2015 alone, approximately 679,100 new cases were diagnosed, and nearly 498,000 deaths occurred due to this disease. The incidence rates continued to be alarming, with the 2020 statistics indicating that 480,000 new cases were diagnosed, representing 10.5% of all new cancer cases in China. Additionally, gastric cancer caused 370,000 deaths, accounting for 12.4% of all cancer-related mortalities in the country, thus positioning it as the third deadliest cancer (2).

The nutrition of patients has emerged as a critical prognostic factor for survival (3). Dietary recommendations for cancer survivors, as outlined by the American Cancer Society, stress the importance of a nutrient-rich diet. This diet includes a variety of vegetables and fruits of different colors, whole grains, and excludes red and processed meats, sugar-sweetened beverages, highly processed foods, and refined grains (4). Despite these guidelines, adherence remains low among cancer survivors, including those with gastrointestinal cancers. For example, their consumption of vegetables, unsweetened dairy products, and nuts is nearly 50% below the recommended levels, and many regularly consume at least one serving of unhealthy food daily (5).

The Knowledge, Attitude, and Practice (KAP) model is foundational in public health research, positing that individual behaviors are significantly influenced by one’s knowledge and attitudes. This model is extensively used to assess health-related behaviors through KAP surveys, which evaluate not only knowledge but also risk perception and behavioral practices (6–8). In the context of gastric cancer, nutritional status is a critical determinant of patient prognosis. In China, where there is a strong tradition of family involvement in patient care, family members often serve as primary caregivers. Consequently, their behaviors and decisions have a direct impact on the dietary habits and nutritional intake of the patients. However, studies have consistently shown low adherence to nutritional guidelines among cancer survivors, underscoring the need to enhance nutritional knowledge and support among family caregivers. By exploring the KAP of family members regarding nutritional care, effective educational and intervention strategies can be designed to improve the overall treatment outcomes and quality of life for gastric cancer patients. Family members play an essential role in cancer management, particularly in fostering adherence to healthy dietary practices and providing daily support.

Therefore, this study aimed to investigate the KAP of family members concerning the nutritional care for gastric cancer patients, highlighting their pivotal role in enhancing patient care and managing treatment outcomes. Although previous KAP studies have examined caregivers’ knowledge, attitudes, and practices in other cancer types such as breast and colorectal cancer (9, 10), research specifically addressing gastrointestinal tumors remains limited. In particular, gastric cancer presents unique nutritional challenges due to surgical alterations to the digestive tract and high risk of cachexia, making caregiver knowledge and involvement even more critical. To our knowledge, this study is one of the first to examine KAP among family caregivers of gastric cancer patients, thus providing novel insights into caregiver preparedness and potential areas for intervention in this specific cancer population. This is consistent with regional findings (11), who investigated knowledge, attitudes and practices among primary caregivers of gastric cancer patients after gastrectomy in China, and found significant gaps particularly in nutritional practice.

## Methods

### Study design and participants

This cross-sectional study was conducted between January 23th to May 7th 2024 at our Hospital, targeting the family members of gastric cancer patients. Inclusion criteria: (1) At least one family member with gastric cancer. (2) Be 18 years of age and above with basic reading and comprehension skills. (3) Willing to participate in the survey and able to provide the necessary personal information and health information of family members. The study obtained ethical approval from our Hospital Institutional Review Board and obtained informed consent from all participants.

### Questionnaire

The design of the questionnaire was guided by established guidelines and informed by previous KAP studies in oncology and nutrition. The initial draft was reviewed by a team of three researchers with clinical experience in gastroenterology and questionnaire development. Although no additional external expert panel was consulted during this phase, care was taken to align the items with relevant literature and practical clinical considerations. A pilot study involving 40 family caregivers (65% female; mean age: 42.1 years) of gastric cancer patients was conducted to assess clarity and applicability. Based on pilot feedback regarding wording and length, minor adjustments were made to improve readability. The reliability of the questionnaire was found to be high, with a Cronbach’s alpha of 0.958 and a Kaiser-Meyer-Olkin (KMO) measure of 0.9526 for the overall scale. Content validity of the questionnaire was evaluated by an expert panel consisting of three gastroenterology clinicians and two nursing specialists. The Item-level CVI (I-CVI) ranged from 0.83 to 1.00, and the Scale-level CVI (S-CVI/Ave) was 0.92, indicating excellent content validity. Construct validity was confirmed using CFA based on the three-factor KAP structure. Model fit indices demonstrated acceptable construct validity (CMIN/DF = 3.541, RMSEA = 0.079, IFI = 0.867, TLI = 0.856, CFI = 0.866), and all factor loadings were significant (*p* < 0.001) (Supplementary Figure S1; Supplementary Table S1). The KMO value was 0.953 (*p* < 0.001), supporting the suitability of the data for factor analysis.

The final version of the questionnaire, written in Chinese, encompassed four dimensions and comprised a total of 44 items (see Supplementary Appendix 1 for details). The basic information dimension included 17 items; the knowledge dimension contained 10 items, with sub-items distributed as follows: item 1 included 5 sub-items, item 2 comprised 2 sub-items, and item 10 included 5 sub-items. There were 8 items in the attitude dimension and 9 in the practice dimension. The scoring system was derived from the options and quantity of items selected: in the knowledge dimension, “Very Familiar” was awarded 2 points, “Heard of” 1 point, and “Unclear” 0 points, allowing for a score range of 0–38 points. Both the attitude and practice dimensions utilized a five-point Likert scale, from “Very Positive” (5 points) to “Very Negative” (1 point). Specifically, in the attitude dimension, items 1 and 3–8 were scored as *a* = 5, *b* = 4, *c* = 3, *d* = 2, *e* = 1; item 2 was reversed, scored as *a* = 1, *b* = 2, *c* = 3, *d* = 4, *e* = 5, resulting in a score range of 8–40 points. In the practice dimension, items 1–9 were scored as *a* = 5, *b* = 4, *c* = 3, *d* = 2, *e* = 1, leading to a score range of 16–80 points. Upon completion of the questionnaire, the knowledge scores were categorized as follows: 0–19 points indicated inadequate knowledge, 20–26 points indicated moderate knowledge, and 27–38 points indicated sufficient knowledge. Attitude scores ranged from 8 to 20 points for a negative attitude, 21–28 points for a neutral attitude, and 29–40 points for a positive attitude. Practice scores ranged from 16–40 points for negative practice behavior, 41–56 points for moderate practice behavior, and 57–80 points for positive practice behavior.

### Variable coding

Sociodemographic variables included age, gender, education level, marital status, family income, and prior caregiving training. For the purpose of regression and SEM analyses, family income was coded in ascending order (1 = ≥15,000 CNY/month, 2 = 8,000–14,999 CNY/month, 3 = 4,000–7,999 CNY/month, 4 = <4,000 CNY/month), such that a higher numerical value represents lower income. Caregiving training was coded as 0 = “received prior training” and 1 = “no prior training.” This coding approach explains the negative *β* coefficients for these variables in the SEM, indicating that lower income (or lack of training) is associated with lower scores in KAP dimensions.

### Questionnaire distribution and quality control

Family members of gastric cancer patients were consecutively recruited using convenience sampling from the gastroenterology and oncology outpatient departments and inpatient wards at a tertiary hospital between January and May 2024. Caregivers who accompanied patients to clinical appointments during the study period were approached in person by trained research staff and were invited to participate if they had access to a smartphone and were willing to complete the electronic survey. The QR code was generated by the online platform Sojump,^1^ and the respondents completed the questionnaire by scanning the code via WeChat. Before answering the questions, participants were required to click the option “I agree to participate in this study” at the beginning of the e-questionnaire. All data were collected anonymously, and to prevent duplication, IP restriction was applied, allowing only one completion of the survey from a single IP address. To ensure data quality and representativeness within the institution, we included caregivers across different departments and time points, and excluded responses with completion times under 90 s or with identical answers across KAP sections. Research assistants were also available to explain difficult points in the questionnaire to participants when necessary.

### Statistical analysis

**Figure 1:**
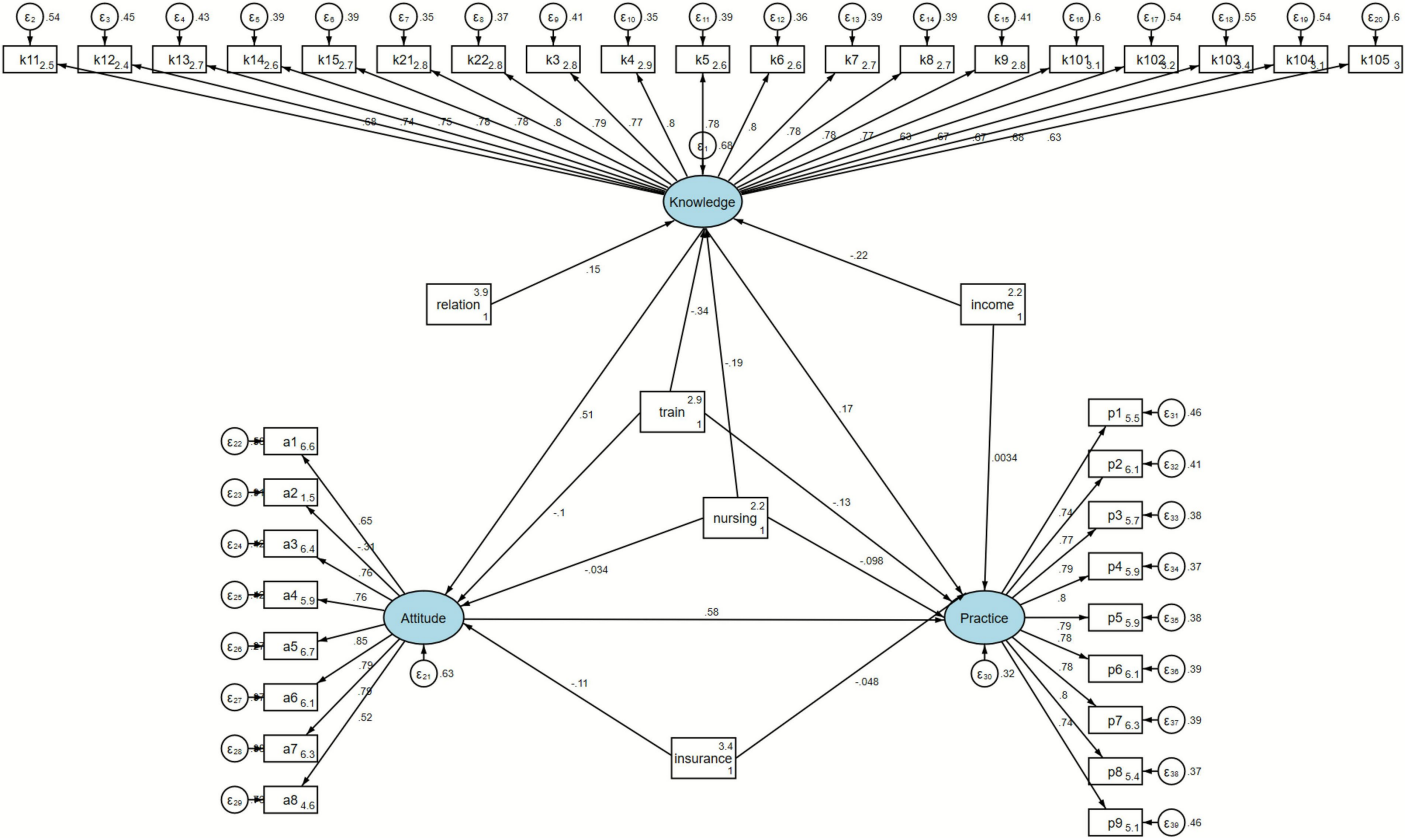
SEM model-path analysis.

Data analysis was conducted using SPSS 22.0 (IBM, Armonk, NY, United States). Continuous variables (KAP scores) that exhibited a normal distribution were presented as means ± standard deviations (SD), including their maximum and minimum values. Categorical data for different demographic characteristics and responses to each questionnaire item were expressed as n (%). For the comparison of KAP dimension scores, continuous variables were initially tested for normality. Variables that were normally distributed were presented as mean ± SD, and comparisons between two groups were conducted using the *t*-test. Non-normally distributed variables were presented as median (range), and the Mann–Whitney U test was employed for comparisons between two groups. For analyses involving three or more groups where continuous variables were normally distributed and variances were equal, an ANOVA was utilized for comparisons. Pearson correlation analysis was employed to assess the relationships among the three KAP dimensions. Structural equation modeling (SEM) was used to explore the relationships and pathways between demographic characteristics and KAP scores. Variables showing statistical significance in descriptive analyses were included in the SEM, under the assumption that these variables influenced the corresponding KAP dimensions. Structural equation modeling (SEM) was conducted using AMOS 26.0 to examine the hypothesized relationships among knowledge, attitudes, and practices based on the KAP framework. According to this theory, knowledge affects attitudes, which in turn influence behavioral practices. Variables showing statistical significance in descriptive analyses were included in the SEM as independent variables to explore their indirect effects on practices via knowledge and attitudes. A conceptual model diagram illustrating the hypothesized pathways has been included as Figure 1. Model fit was assessed using the following indices: root mean square error of approximation (RMSEA), standardized root mean square residual (SRMR), CFI, and TLI. The model demonstrated acceptable fit (RMSEA = 0.081, SRMR = 0.055, CFI = 0.822, TLI = 0.810), meeting the recommended thresholds for structural models (RMSEA < 0.08; CFI & TLI > 0.80). Although the RMSEA value (0.081) was slightly above the recommended cutoff of 0.08, values between 0.08 and 0.10 are considered acceptable in complex behavioral models, and overall model fit indices supported the adequacy of the model. A two-sided *p*-value less than 0.05 was considered statistically significant.

## Results

Initially, a total of 450 responses were collected. After excluding: 1. 6 cases of non-informed consent; 2. 38 cases with response times less than 80 s; The final valid dataset consisted of 406 cases. Among the 406 family members of gastric cancer patients who participated in this study, 243(59.9) were children of the patients, 216(53.2) were female, 221(54.4) were employed, and 194(47.8) had average monthly income per capita of 2,000–5,000 Yuan. Meanwhile, 160(39.4) patients had been diagnosed with gastric cancer for less than 6 months, 254(62.6) were receiving nutritional care for gastric cancer, 221(54.4) had participated in nutritional care education or training, 144(35.5) had underlying or chronic diseases, and 178(43.8) were in stage III-IV of gastric cancer. The median (25.75%) knowledge, attitude, and practice scores were 27.00 (19.00, 33.00), 31.00 (30.00, 36.00), and 36.00 (34.00, 42.00), separately. Analyses of demographic characteristics found that participants’ knowledge, attitude, and practice scores varied across smoking habit (*p* = 0.007, *p* = 0.006, *p* = 0.024), medical insurance or other commercial insurance (*p* = 0.005, *p* = 0.004, *p* < 0.001), whether receive nutritional care for gastric cancer (*p* < 0.001, *p* < 0.001, *p* < 0.001), and whether participated in education or training on cancer nutrition care (*p* < 0.001, *p* < 0.001, *p* < 0.001). Meanwhile, participants’ knowledge scores were more likely to vary depending on: relationship with the patient (*p* < 0.001), employment status (*p* = 0.007), average monthly income per capita (*p* < 0.001), and stage of gastric cancer in the patient (*p* < 0.001). Participants’ attitude scores were more likely to vary depending on: education (*p* = 0.023) and drinking habit (*p* = 0.011). Participants’ practice scores were more likely to vary depending on: relationship with the patient (*p* = 0.019) average monthly income per capita (*p* = 0.027), drinking habit (*p* = 0.018), and stage of gastric cancer in the patient (*p* < 0.001) (Table 1).

**Table 1:** Demographic characteristics and KAP scores.

Characteristics	N(%)	Knowledge	*P*	Attitude	*P*	Practice	*P*
		Median [25.75%]		Median [25.75%]		Median [25.75%]	
Total score	406(100.0)	27.00 [19.00, 33.00]		31.00 [30.00, 36.00]		36.00 [34.00, 42.00]	
Relationship with the patient is			<0.001		0.537		0.019
Parents	32(7.9)	18.00 [11.25, 22.00]		31.00 [24.00, 36.00]		34.00 [27.00, 40.25]	
Spouse	131(32.3)	26.00 [20.00, 32.50]		32.00 [30.00, 36.00]		38.00 [34.00, 43.00]	
Children	243(59.9)	28.00 [19.00, 34.00]		31.00 [30.00, 36.00]		36.00 [34.00, 41.00]	
Age			0.938		0.419		0.342
18–34 years old	56(13.8)	26.00 [19.00, 33.00]		30.00 [30.00, 36.00]		36.50 [33.00, 40.00]	
35–44 years old	144(35.5)	26.50 [18.00, 32.00]		32.00 [30.00, 36.00]		36.00 [33.00, 40.00]	
45–54 years old	107(26.4)	27.00 [18.00, 35.00]		32.00 [30.00, 36.00]		36.00 [34.50, 44.50]	
55–64 years old	65(16.0)	27.00 [21.00, 30.00]		33.00 [30.00, 35.00]		38.00 [35.00, 43.00]	
65 years old and above	34(8.4)	23.00 [17.00, 34.50]		31.00 [30.00, 36.00]		36.00 [31.25, 44.50]	
Gender			0.712		0.452		0.625
Male	190(46.8)	27.00 [19.00, 34.00]		31.00 [30.00, 36.00]		36.50 [34.00, 41.00]	
Female	216(53.2)	26.50 [18.75, 33.00]		32.00 [30.00, 36.00]		36.00 [34.00, 42.25]	
Education			0.122		0.023		0.896
Primary school and below	21(5.2)	22.00 [12.00, 29.00]		32.00 [30.00, 34.00]		36.00 [35.00, 41.00]	
Junior high school	106(26.1)	24.00 [18.00, 31.00]		31.00 [29.25, 34.75]		36.00 [33.00, 40.00]	
High school/technical school	135(33.3)	27.00 [19.00, 35.00]		31.00 [30.00, 35.00]		37.00 [35.00, 41.00]	
College/undergraduate	132(32.5)	27.00 [20.00, 34.25]		32.50 [30.00, 36.00]		36.00 [34.00, 44.00]	
Master’s degree and above	12(3.0)	28.50 [17.75, 38.00]		30.50 [27.75, 36.00]		36.50 [30.75, 45.00]	
Ethnicity			0.162		0.872		0.127
Han	351(86.5)	27.00 [19.00, 34.00]		31.00 [30.00, 36.00]		36.00 [34.00, 42.50]	
Minority	55(13.5)	26.00 [16.00, 30.00]		32.00 [30.00, 35.50]		36.00 [31.00, 41.00]	
Employment status			0.007		0.680		0.090
Employed	221(54.4)	28.00 [20.00, 35.00]		32.00 [30.00, 36.00]		37.00 [34.00, 43.00]	
Unemployed	10(2.5)	29.00 [12.25, 36.75]		33.00 [31.00, 36.00]		40.00 [36.25, 43.75]	
Retired	60(14.8)	26.50 [20.00, 30.00]		32.00 [30.00, 35.25]		39.00 [35.75, 43.00]	
Self-employed	68(16.7)	23.00 [15.00, 31.00]		31.00 [30.00, 35.25]		36.00 [32.00, 39.25]	
Other	47(11.6)	21.00 [17.00, 26.00]		31.00 [30.00, 36.00]		36.00 [30.50, 41.00]	
Monthly household income			<0.001		0.086		0.027
<2,000	36(8.9)	23.00 [12.00, 28.00]		31.50 [30.00, 34.25]		36.00 [33.75, 40.25]	
2,000–5,000	194(47.8)	28.00 [21.00, 37.00]		32.00 [30.00, 36.00]		37.00 [34.00, 45.00]	
5,000–10,000	121(29.8)	27.00 [19.00, 32.00]		31.00 [30.00, 35.00]		37.00 [36.00, 41.00]	
>10,000	22(5.4)	25.50 [19.50, 31.00]		31.00 [30.00, 36.00]		36.00 [31.25, 42.50]	
Prefer not to disclose	33(8.1)	10.00 [7.00, 17.00]		30.00 [27.00, 36.00]		34.00 [31.00, 37.00]	
Marital status			0.386		0.429		0.167
Unmarried	26(6.4)	21.50 [17.25, 30.75]		30.00 [30.00, 35.75]		36.00 [30.50, 40.25]	
Married	367(90.4)	27.00 [19.00, 33.00]		32.00 [30.00, 36.00]		36.00 [34.00, 42.00]	
Divorced	13(3.2)	31.00 [23.00, 32.00]		31.00 [28.00, 34.00]		39.00 [38.00, 41.00]	
Ever given birth			0.496		0.112		0.066
Yes	361(88.9)	27.00 [19.00, 33.00]		32.00 [30.00, 36.00]		37.00 [34.00, 42.00]	
No	45(11.1)	23.00 [19.00, 33.00]		30.00 [30.00, 35.00]		36.00 [32.00, 39.00]	
Smoking			0.007		0.006		0.024
Never smoked	265(65.3)	27.00 [19.00, 35.00]		32.00 [30.00, 36.00]		37.00 [34.00, 43.00]	
Used to smoke	85(20.9)	27.00 [22.00, 30.00]		31.00 [30.00, 34.00]		36.00 [34.00, 40.00]	
Still smoke now	56(13.8)	21.00 [11.25, 30.25]		30.00 [29.00, 34.25]		36.00 [31.00, 41.00]	
Drinking			0.076		0.011		0.018
Never drank alcohol	236(58.1)	27.00 [19.00, 35.25]		32.00 [30.00, 36.00]		37.00 [34.00, 45.00]	
Used to drink alcohol	101(24.9)	25.00 [19.00, 30.00]		31.00 [30.00, 34.00]		36.00 [34.00, 39.00]	
Current drinker	69(17.0)	25.00 [16.00, 32.00]		31.00 [30.00, 35.00]		36.00 [32.00, 40.00]	
Medical/commercial insurance			0.005		0.004		<0.001
Yes	353(86.9)	27.00 [19.00, 33.00]		32.00 [30.00, 36.00]		37.00 [35.00, 43.00]	
No	53(13.1)	20.00 [14.00, 31.00]		30.00 [28.00, 34.00]		34.00 [29.00, 36.00]	
Gastric cancer duration			0.308		0.624		0.468
Within 6 months	160(39.4)	27.00 [18.00, 32.00]		31.00 [30.00, 36.00]		36.00 [34.00, 42.00]	
Half a year—1 year	154(37.9)	26.00 [19.00, 33.00]		32.00 [30.00, 36.00]		37.00 [34.00, 42.75]	
1 ~ 2 years	57(14.0)	29.00 [19.00, 37.00]		31.00 [30.00, 36.00]		36.00 [32.00, 43.00]	
2 years and above	35(8.6)	23.00 [18.00, 32.00]		31.00 [30.00, 35.50]		36.00 [33.00, 40.00]	
Nutritional care			<0.001		<0.001		<0.001
Yes	254(62.6)	28.50 [21.00, 38.00]		33.00 [30.00, 36.00]		39.00 [36.00, 45.00]	
No	110(27.1)	24.00 [17.00, 30.00]		30.00 [30.00, 33.75]		35.00 [29.00, 36.00]	
Uncertain	42(10.3)	18.00 [11.25, 22.00]		30.00 [28.00, 32.75]		35.00 [28.50, 37.00]	
Cancer nutrition training			<0.001		<0.001		<0.001
Yes	221(54.4)	30.00 [24.00, 38.00]		34.00 [30.00, 36.00]		40.00 [36.00, 45.00]	
No	185(45.6)	20.00 [12.00, 27.00]		30.00 [29.00, 32.00]		35.00 [30.00, 37.00]	
Underlying/chronic diseases			0.811		0.937		0.242
Yes	262(64.5)	26.00 [18.00, 35.00]		31.00 [30.00, 36.00]		36.00 [34.00, 42.00]	
No	144(35.5)	27.00 [20.00, 31.25]		32.00 [30.00, 35.00]		38.00 [34.00, 41.25]	
Hypertension	71(17.5)	25.00 [18.00, 31.00]		32.00 [30.00, 36.00]		38.00 [34.00, 43.00]	
Hyperlipidemia	17(4.2)	29.00 [27.00, 32.00]		31.00 [30.00, 34.00]		39.00 [36.00, 40.00]	
Diabetes	37(9.1)	28.00 [24.00, 30.00]		32.00 [30.00, 35.00]		39.00 [35.00, 43.00]	
Tumor	3(0.7)	29.00 [28.00, 29.50]		33.00 [32.00, 35.50]		35.00 [34.00, 37.00]	
Hepatitis	4(1.0)	21.50 [18.00, 25.75]		30.50 [29.25, 32.25]		38.50 [36.00, 41.25]	
Asthma/chronic obstructive pulmonary disease (COPD)	4(1.0)	24.00 [16.00, 30.75]		33.00 [30.00, 34.50]		36.00 [31.75, 40.75]	
Other (e.g., respiratory, digestive system diseases)	8(2.0)	23.00 [14.25, 33.00]		31.00 [30.00, 35.00]		35.50 [28.50, 39.25]	
Stage of gastric cancer			<0.001		0.152		<0.001
Stage I-IV	76(18.7)	29.00 [22.75, 38.00]		32.00 [30.00, 36.00]		40.00 [36.00, 45.00]	
Stages I-II are early-stage gastric cancer	57(14.0)	27.00 [19.00, 38.00]		32.00 [30.00, 36.00]		38.00 [34.00, 45.00]	
Stages III-IV are advanced stages	178(43.8)	27.00 [20.00, 32.75]		32.00 [30.00, 36.00]		36.00 [34.00, 40.00]	
Uncertain	95(23.4)	19.00 [9.00, 25.50]		30.00 [28.00, 36.00]		36.00 [31.00, 41.00]	

The distribution of knowledge dimensions shown that the three questions with the highest number of participants choosing the “Unclear” option were “Difficulty in intake caused by mechanical factors.” (K1.2) with 23.2%, “Factors combined with increased catabolism, such as infection or surgical treatment. Gastric cancer patients who also have smoking and drinking habits are prone to local infections when their granulocyte count drops during concurrent radiotherapy and chemotherapy.” (K1.4) with 21.2%, and “During gastric cancer surgery, cutting the vagus nerve or pylorus removal can accelerate postoperative gastric emptying, increase gastric juice loss, and cause pancreatic and biliary dysfunction, leading to digestion and absorption disorders of fats, proteins, and carbohydrates. Increased excretion of carbohydrates, fats, and proteins in feces after surgery can lead to postoperative malnutrition in patients. Therefore, high-calorie, high-protein diets should be supplemented.” (K6) with 20.7% (Table 2).

**Table 2:** Distribution of knowledge dimension responses.

Knowledge	Very familiar	Heard of	Unclear
1.1 Anorexia and depression caused by the disease itself lead to reduced food intake. Among all tumors, gastric cancer has the highest incidence of anorexia and early satiety.	150(36.9%)	182(44.8%)	74(18.2%)
1.2 Difficulty in intake caused by mechanical factors.	146(36%)	166(40.9%)	94(23.2%)
1.3 Absorption and digestive disorders caused by the toxicity of chemotherapy drugs.	164(40.4%)	175(43.1%)	67(16.5%)
1.4 Factors combined with increased catabolism, such as infection or surgical treatment. Gastric cancer patients who also have smoking and drinking habits are prone to local infections when their granulocyte count drops during concurrent radiotherapy and chemotherapy.	155(38.2%)	165(40.6%)	86(21.2%)
1.5 Specific effects of gastric surgery: Among all gastrointestinal surgeries, gastric surgery has the most complications, the greatest impact on nutrition and metabolism, and the longest duration. Metabolic changes and absorption disorders caused by gastric resection and diversion should be given due attention, such as absorption disorders and deficiencies of iron, calcium, vitamin A, vitamin B12, and vitamin D caused by gastric juice loss, and digestion and absorption disorders of fats, proteins, and carbohydrates.	175(43.1%)	162(39.9%)	69(17%)
2.1 Weakening of the efficacy of radiotherapy and chemotherapy, increased risk of adverse drug reactions, and reduced skeletal muscle mass and function.	153(37.7%)	190(46.8%)	63(15.5%)
2.2 Increased chances of postoperative complications and nosocomial infections, prolonged hospital stay, increased incidence of complications and mortality, deterioration of patients’ quality of life, and increased medical expenses.	171(42.1%)	170(41.9%)	65(16%)
3. The approaches to nutritional therapy for gastric cancer patients include enteral nutrition (oral, tube feeding) and parenteral nutrition (intravenous).	168(41.4%)	175(43.1%)	63(15.5%)
4. After gastric cancer surgery, the stomach’s volume significantly decreases, and overeating can lead to symptoms such as bloating and nausea. Therefore, consuming smaller, more frequent meals can alleviate bloating symptoms.	181(44.6%)	168(41.4%)	57(14%)
5. After gastric cancer surgery, the stomach’s grinding function is partially or completely lost, so the chewing function of the teeth needs to partially substitute for the stomach’s role. Patients should eat slowly after surgery to prevent rapid food expulsion, which affects digestion and absorption, and to prevent dumping syndrome. Patients should rest in a semi-recumbent position after meals to prolong the emptying time of food for complete digestion and absorption.	159(39.2%)	171(42.1%)	76(18.7%)
6. During gastric cancer surgery, cutting the vagus nerve or pylorus removal can accelerate postoperative gastric emptying, increase gastric juice loss, and cause pancreatic and biliary dysfunction, leading to digestion and absorption disorders of fats, proteins, and carbohydrates. Increased excretion of carbohydrates, fats, and proteins in feces after surgery can lead to postoperative malnutrition in patients. Therefore, high-calorie, high-protein diets should be supplemented.	160(39.4%)	162(39.9%)	84(20.7%)
7. The highest level of nutrition is balance, and special attention should be paid to deficiencies in vitamins and trace elements after gastric cancer surgery. Patients can consume moderate amounts of liver, red meat, seafood, milk, various soy products, and dairy products.	180(44.3%)	154(37.9%)	72(17.7%)
8. Patients can engage in appropriate exercises when their bodies have not fully recovered after gastric cancer surgery, such as walking, jogging, Tai Chi, and aerobics, to activate muscles, enhance physical strength, promote gastrointestinal motility, help digest food, improve appetite, and increase food intake.	176(43.3%)	156(38.4%)	74(18.2%)
9. Prolonged negative emotions such as loneliness, sadness, and despair can cause neuroendocrine disorders, weaken immune surveillance function, and lead to sudden proliferation of cancer cells. Therefore, maintaining a positive attitude is crucial for gastric cancer patients. When patients feel low or in poor mental condition, they should talk to family or friends, build confidence in overcoming the disease, and regulate their emotions through reading, watching TV, participating in recreational activities, and listening to music.	194(47.8%)	146(36%)	66(16.3%)
10.1 Medication should be taken at fixed times and in accordance with medical advice.	247(60.8%)	118(29.1%)	41(10.1%)
10.2 Tumor drugs should not be arbitrarily reduced in dosage.	265(65.3%)	102(25.1%)	39(9.6%)
10.3 During treatment, adverse drug reactions should be closely monitored.	270(66.5%)	105(25.9%)	31(7.6%)
10.4 Drugs should be stored separately, and antitumor drugs should generally be stored in a light-resistant, dry place away from heat sources, with a temperature below 25 degrees Celsius being suitable. If there are children at home, special attention should be paid to keeping the drugs out of reach.	247(60.8%)	118(29.1%)	41(10.1%)
10.5 When taking analgesics, follow the three principles of medication: timely oral administration, administration on schedule, and administration according to the stepwise principle.	238(58.6%)	127(31.3%)	41(10.1%)

Responses to the attitudinal dimension showed that 31.5% strongly agreed and 35.7% agreed that they felt overwhelmed and lacked confidence and patience in nutritional care for gastric cancer patients (A2). Regarding the statement that medical institutions do not provide enough education on nutritional care for gastric cancer patients (A8), 19.5% were neutral and 7.4% disagreed (Table 3).

**Table 3:** Distribution of attitude dimension responses.

Attitude	Strongly agree	Agree	Neutral	Disagree	Strongly disagree
1. I believe that nutritional care for gastric cancer patients is key to improving their quality of life. (P)	200(49.3%)	165(40.6%)	32(7.9%)	4(1%)	5(1.2%)
2. I find it challenging to provide nutritional care for gastric cancer patients, and I lack confidence and patience. (N)	128(31.5%)	145(35.7%)	68(16.7%)	47(11.6%)	18(4.4%)
3. I believe that balanced nutrition for gastric cancer patients is one of the most important aspects of nutritional care that family members should pay attention to. (P)	184(45.3%)	171(42.1%)	42(10.3%)	2(0.5%)	7(1.7%)
4. I believe that family members of gastric cancer patients need professional training in nutritional care. (P)	168(41.4%)	175(43.1%)	49(12.1%)	5(1.2%)	9(2.2%)
5. To improve the effectiveness of nutritional care, I believe that family members need to pay attention to the patient’s psychological and emotional wellbeing. (P)	191(47%)	176(43.3%)	29(7.1%)	3(0.7%)	7(1.7%)
6. I believe that regular follow-ups help to adjust nutritional care strategies in a timely manner for greater benefits. (P)	178(43.8%)	181(44.6%)	32(7.9%)	5(1.2%)	10(2.5%)
7. I believe that patients often need encouragement from healthcare professionals and family members to boost their confidence and patience in nutritional therapy. (P)	177(43.6%)	187(46.1%)	29(7.1%)	4(1%)	9(2.2%)
8. I believe that medical institutions do not provide enough education on nutritional care for gastric cancer patients. (P)	144(35.5%)	144(35.5%)	79(19.5%)	30(7.4%)	9(2.2%)

Responses to the practice dimension showed that 7.1% rarely and 3% never participated in any related educational activities (P9), 5.4% rarely and 1.7% never took the initiative to seek professional medical advice to make timely adjustments to nutritional care strategies (P3), and 4.9% rarely and 3% never discussed the process of developing a nutritional care plan for the patient with the doctor and dietitian (P8) (Table 4).

**Table 4:** Distribution of practice dimension responses.

Practice	Always	Often	Sometimes	Rarely	Never
1. I will proactively learn about nutritional care for gastric cancer patients. (P)	137(33.7%)	170(41.9%)	69(17%)	20(4.9%)	10(2.5%)
2. I will strictly control the patient’s diet to achieve balanced nutrition as much as possible. (P)	139(34.2%)	176(43.3%)	75(18.5%)	10(2.5%)	6(1.5%)
3. When encountering problems, I will seek professional medical advice proactively to adjust the nutritional care strategy in a timely manner. (P)	143(35.2%)	164(40.4%)	70(17.2%)	22(5.4%)	7(1.7%)
4. I will actively observe any adverse reactions during the patient’s home treatment process. (P)	151(37.2%)	178(43.8%)	53(13.1%)	14(3.4%)	10(2.5%)
5. I will encourage and accompany the patient to engage in moderate exercise. (P)	158(38.9%)	161(39.7%)	65(16%)	16(3.9%)	6(1.5%)
6. I will always pay attention to the patient’s psychological fluctuations and care for their emotions. (P)	162(39.9%)	177(43.6%)	47(11.6%)	13(3.2%)	7(1.7%)
7. I will schedule regular follow-up appointments with the patient. (P)	175(43.1%)	169(41.6%)	47(11.6%)	8(2%)	7(1.7%)
8. I will actively participate in the process of developing nutritional care plans for patients with doctors and dietitians. (P)	162(39.9%)	145(35.7%)	67(16.5%)	20(4.9%)	12(3%)
9. I will actively participate in any educational activities on nutritional care for gastric cancer patients organized by medical institutions. (P)	150(36.9%)	140(34.5%)	75(18.5%)	29(7.1%)	12(3%)

Correlation analysis showed that there were significant positive correlations between knowledge and attitude (*r* = 0.503, *p* < 0.001) as well as practice (*r* = 0.598, *p* < 0.001). Also, there was a correlation between attitude and practice (*r* = 0.634, *p* < 0.001) (Table 5).

**Table 5:** Correlation analysis.

Variables	Knowledge	Attitude	Practice
Knowledge	1.000		
Attitude	0.503 (*P* < 0.001)	1.000	
Practice	0.598 (*P* < 0.001)	0.634 (*P* < 0.001)	1.000

The hypothesized SEM model demonstrated acceptable fit, with the following indices: RMSEA = 0.081, SRMR = 0.055, CFI = 0.822, and TLI = 0.810, all meeting recommended thresholds (Table 6).

**Table 6:** Model fit.

Indicators	Reference	Results
RMSEA	<0.08 good	0.081
SRMR	<0.08 good	0.055
TLI	>0.8 good	0.810
CFI	>0.8 good	0.822

Knowledge was directly influenced by the caregiver–patient relationship (*β* = 3.50, *p* < 0.001), income (*β* = −4.98, *p* < 0.001), caregiving role (nursing; *β* = −3.73, *p* < 0.001), and prior training (*β* = −6.25, *p* < 0.001). Attitude was directly influenced by knowledge (*β* = 7.78, *p* < 0.001), caregiver–patient relationship (*β* = 3.30, *p* = 0.001), income (*β* = −4.44, *p* < 0.001), medical insurance type (*β* = −2.44, *p* = 0.015), caregiving role (*β* = −2.31, *p* = 0.021), and training (*β* = −4.63, *p* < 0.001). Practice was directly influenced by knowledge (*β* = 8.10, *p* < 0.001), attitude (*β* = 9.46, *p* < 0.001), caregiver–patient relationship (*β* = 3.32, *p* = 0.001), income (*β* = −2.58, *p* = 0.010), medical insurance type (*β* = −2.73, *p* = 0.006), caregiving role (*β* = −3.94, *p* < 0.001), and training (*β* = −6.38, *p* < 0.001) (Table 7; Figure 1).

**Table 7:** Results of SEM path analysis.

Indicators	Estimate	*P* > |z|
Knowledge		
Relation	3.5	<0.001
Income	−4.98	<0.001
Nursing	−3.73	<0.001
Train	−6.25	<0.001
Attitude		
Knowledge	7.78	<0.001
Relation	3.3	0.001
Income	−4.44	<0.001
Insurance	−2.44	0.015
Nursing	−2.31	0.021
Train	−4.63	<0.001
Practice		
Knowledge	8.1	<0.001
Attitude	9.46	<0.001
Relation	3.32	0.001
Income	−2.58	0.010
Insurance	−2.73	0.006
Nursing	−3.94	<0.001
Train	−6.38	<0.001

Coding of categorical variables was as follows: Caregiver–patient relationship: 1 = Parent; 2 = Spouse; 3 = Child. Monthly income (CNY): 1 = <2,000; 2 = 2,000–5,000; 3 = 5,000–10,000; 4 = Prefer not to disclose. Caregiving experience: 1 = Yes; 2 = No; 3 = Uncertain. Training received: 1 = Yes; 2 = No. Negative β coefficients for income, caregiving experience, and training indicate that higher numerical coding values—which represent lower income, lack of caregiving experience, and absence of training—are associated with lower scores in the corresponding KAP dimensions.

Mediation analysis further revealed that knowledge mediated the effects of caregiver–patient relationship (*β* = 0.078, *p* = 0.001), income (*β* = −0.113, *p* < 0.001), caregiving role (*β* = −0.098, *p* = 0.001), and training (*β* = −0.174, *p* < 0.001) on attitude. Both knowledge and attitude mediated the effects of caregiver–patient relationship (*β* = 0.071, *p* = 0.001), income (*β* = −0.104, *p* < 0.001), medical insurance type (*β* = −0.063, *p* = 0.016), caregiving role (*β* = −0.110, *p* = 0.005), and training (*β* = −0.218, *p* < 0.001) on practice (Table 8).

**Table 8:** Mediation analysis.

Model paths		Total effects		Direct effect		Indirect effect	
		β (95% CI)	*P*	β (95% CI)	*P*	β (95% CI)	*P*
Knowledge							
	Relation	0.151 (0.066, 0.236)	<0.001	0.151 (0.066, 0.236)	<0.001		
	Income	−0.220 (−0.307, −0.134)	<0.001	−0.220 (−0.307, −0.134)	<0.001		
	Nursing	−0.191 (−0.291, −0.090)	<0.001	−0.191 (−0.291, −0.090)	<0.001		
	Train	−0.339 (−0.446, −0.233)	<0.001	−0.339 (−0.446, −0.233)	<0.001		
Attitude							
	Knowledge	0.514 (0.384, 0.643)	<0.001	0.514 (0.384, 0.643)	<0.001		
	Relation	0.078 (0.031, 0.124)	0.001			0.078 (0.031, 0.124)	0.001
	Income	−0.113 (−0.163, −0.063)	<0.001			−0.113 (−0.163, −0.063)	<0.001
	Insurance	−0.108 (−0.195, −0.021)	0.015	−0.108 (−0.195, −0.021)	0.015		
	Nursing	−0.132 (−0.244, −0.020)	0.021	−0.034 (−0.135,0.068)	0.512	−0.098 (−0.153, −0.043)	0.001
	Train	−0.274 (−0.390, −0.158)	<0.001	−0.100 (−0.208, 0.009)	0.071	−0.174 (−0.239, −0.109)	<0.001
Practice							
	Knowledge	0.472 (0.358, 0.586)	<0.001	0.173 (0.077, 0.268)	<0.001	0.300 (0.215, 0.384)	<0.001
	Attitude	0.584 (0.463, 0.704)	<0.001	0.584 (0.463, 0.704)	<0.001		
	Relation	0.071 (0.029, 0.114)	0.001			0.071 (0.029, 0.114)	0.001
	Income	−0.101 (−0.177, −0.024)	0.010	0.003 (−0.063, 0.070)	0.920	−0.104 (−0.149, −0.059)	<0.001
	Insurance	−0.111 (−0.190, −0.031)	0.006	−0.048 (−0.114, 0.019)	0.161	−0.063 (−0.114, −0.012)	0.016
	Nursing	−0.208 (−0.312, −0.105)	<0.001	−0.099 (−0.177, −0.020)	0.014	−0.110 (−0.186, −0.034)	0.005
	Train	−0.350 (−0.457, −0.242)	<0.001	−0.132 (−0.215, −0.048)	0.002	−0.218 (−0.300, −0.137)	<0.001

Coding of categorical variables was as follows: Caregiver–patient relationship: 1 = Parent; 2 = Spouse; 3 = Child. Monthly income (CNY): 1 = <2,000; 2 = 2,000–5,000; 3 = 5,000–10,000; 4 = Prefer not to disclose. Caregiving experience: 1 = Yes; 2 = No; 3 = Uncertain. Training received: 1 = Yes; 2 = No. Negative β coefficients for income, caregiving experience, and training indicate that higher numerical coding values—which represent lower income, lack of caregiving experience, and absence of training—are associated with lower scores in the corresponding KAP dimensions.

## Discussion

The study highlights that family members generally possess sufficient knowledge and positive attitudes toward the nutritional care of gastric cancer patients, yet these factors do not consistently translate into effective practices. Clinical interventions should focus on enhancing practical applications of nutritional knowledge among family caregivers, potentially through targeted training and support programs that bridge the gap between knowledge and action.

The correlations between knowledge and both attitude and practice, supported by the SEM outcomes, suggest that enhancing knowledge could sequentially improve attitudes and practices. This is consistent with previous research indicating that better-informed caregivers are more likely to adopt positive attitudes and effective practices in patient care (12, 13). Besides, significant differences were observed across various demographic factors such as relationship to the patient, educational level, and income, which also correlated significantly with the KAP scores.

SEM analysis reveals that the nature of the familial relationship directly impacts KAP dimensions. Specifically, spouses, who typically assume primary caregiving roles, exhibit higher knowledge and practice scores. This association highlighted that those who have direct caregiving responsibilities are more likely to acquire and apply healthcare knowledge effectively (14, 15). Lower income groups displayed lower knowledge and practice scores, a pattern supported by SEM, indicating a direct negative effect of lower income on KAP. This likely reflects socioeconomic barriers to accessing healthcare education and resources, as documented in studies linking economic status with health literacy and outcomes (16, 17). Higher educational levels were found to significantly affect attitudes, supported by SEM analyses that highlight education’s role in shaping perceptual and cognitive aspects of care. This aligns with literature indicating that higher education levels can improve health awareness and value orientation toward disease management (18, 19). However, the non-significant impact on knowledge and practices might suggest that educational content may not be adequately targeted toward actionable skills or that other barriers prevent the translation of knowledge into practice.

Moreover, the findings highlight a direct correlation between non-smoking and higher knowledge and attitudes, an observation supported by literature that associates non-smoking with a general proclivity toward healthier lifestyle choices (20, 21). Participants with medical insurance demonstrated higher scores in all KAP aspects. SEM analyses elucidate that insurance facilitates not just access to healthcare but also influences the comprehensive engagement with health-promoting behaviors. This is consistent with studies that discuss how insurance coverage can alleviate financial barriers and enhance patient and caregiver engagement in preventive and continuous care practices (22). Engagement in specialized educational or training programs was a significant predictor of better KAP outcomes in both the statistical and SEM analyses. This finding underscores the effectiveness of targeted educational interventions in bridging the knowledge-practice gap and is supported by educational theory which posits that specific, contextually relevant training enhances both cognitive and behavioral competencies in healthcare settings (23, 24).

In addition to these demographic influences, specific knowledge gaps were identified in items related to postoperative complications (e.g., the effects of gastrectomy on nutrient absorption and metabolism) and the impact of chemotherapy on digestive function (25). These concepts may be perceived as complex or insufficiently addressed during clinical visits, contributing to uncertainty among caregivers. Similar challenges have been reported in studies investigating caregiver education for gastrointestinal cancer patients, where the lack of digestible, actionable information limited caregivers’ ability to support postoperative recovery effectively. This knowledge gap likely contributes to inconsistent practices observed in the study, such as limited engagement in monitoring for adverse reactions or seeking professional nutritional advice. Barriers such as time constraints, limited access to dietitian services, emotional stress, and lack of structured educational resources may further hinder the translation of positive attitudes into consistent practice behavior (26). To address these challenges, integrating multidisciplinary dietary counseling, visual tools, or mobile-based education platforms into routine care could enhance both comprehension and adherence among caregivers (27, 28).

The knowledge dimension reveals varied levels of familiarity with key aspects of nutritional care among caregivers of gastric cancer patients, with significant percentages indicating limited clarity on crucial issues such as the complications of gastric surgery and the impact of chemotherapy on digestion and absorption. Notably, areas such as the specific effects of surgery on metabolism and the role of the vagus nerve in gastric functioning are less understood. This reflects findings from the study by Leonard et al., which highlighted a general gap in caregivers’ medical knowledge, particularly in the complexities of cancer care (29). These findings may reflect the complexity of postoperative physiological changes, which are typically explained using medical terminology that is difficult for non-professional caregivers to understand. Additionally, postoperative consultations often prioritize disease progression and treatment plans, with limited time allocated to detailed nutritional guidance. This may further contribute to the inadequate understanding of key nutritional concepts among caregivers. To address these gaps, targeted educational programs designed specifically for caregivers are recommended. These should include detailed modules on the physiological impacts of gastric cancer treatments, employing visual aids and practical demonstrations to enhance understanding. For example, simplified visual materials illustrating the expected changes in digestion after gastrectomy could be developed. Regular caregiver workshops led by clinical dietitians may support hands-on learning about meal planning and nutritional risk monitoring. Furthermore, establishing community-based support groups would allow caregivers to share experiences, receive peer support, and consult experts in a more relaxed and accessible environment. Furthermore, the development of accessible online resources and mobile applications that provide reliable information and reminders about the specific care requirements following gastric cancer surgery could prove beneficial (30–32).

While the overall attitude toward the importance of nutritional care is positive, there is a notable portion of respondents who express challenges and lack confidence in providing this care. This is particularly evident in the perceived difficulties in managing the nutritional needs of gastric cancer patients. In response, it is advisable to implement support groups and counseling services that address these emotional and psychological barriers. Training sessions led by dietitians and psychologists that focus on building caregiver confidence and patience could be instrumental. Additionally, integrating caregiver feedback into the development of nutritional guidelines could personalize and simplify the care process, making it more manageable for caregivers (33, 34).

The practice dimension indicates a proactive stance in learning and managing nutritional care, yet there are inconsistencies in application, with a minority of caregivers seldom engaging in recommended practices like monitoring for adverse reactions or scheduling regular follow-ups. To enhance practical application, regular audits and feedback sessions could help caregivers assess their adherence to nutritional care plans and identify areas for improvement. Additionally, establishing a partnership with local health centers to provide ongoing support and resources, including regular check-ins by a dietitian, could help maintain a high standard of care (35, 36).

This study has several limitations. First, its cross-sectional design restricts the ability to establish causal relationships between the variables studied and the outcomes observed. Second, the use of convenience sampling limits the generalizability of the findings, as participants were recruited from a single institution and may not represent all family caregivers of gastric cancer patients across different settings. Specifically, this study was conducted in Inner Mongolia, a region with unique socio-economic and cultural characteristics, including lower average household income and distinct dietary customs, which may lead to different levels of caregiver awareness and nutritional practices compared to other regions in China. Additionally, recruitment through clinical settings may have introduced selection bias, as caregivers more actively involved in patient care or those with higher health awareness may have been more likely to participate. Future studies should consider employing random sampling strategies across multiple centers to enhance representativeness and account for regional variations. Third, the reliance on self-reported data to assess knowledge, attitudes, and practices may introduce recall bias and social-desirability bias, as participants might overestimate their knowledge or report more favorable behaviors. Future studies could mitigate this by incorporating objective measures, such as dietary logs, patient nutritional assessments, or observational data on caregiving practices, to validate the accuracy of self-reported indicators.

## Conclusion

In conclusion, this study suggests that while many family caregivers of gastric cancer patients possess adequate knowledge and display positive attitudes toward nutritional care, their actual practices remain insufficient. Given the observed associations rather than causal relationships, tailored educational interventions may help improve caregiving practices. Practical strategies could include structured nutrition workshops led by clinical dietitians, the provision of culturally appropriate nutrition manuals, and regular counseling sessions that address specific caregiving challenges. Such approaches may support caregivers in translating positive attitudes into effective nutritional care behaviors that benefit patient outcomes.

## Data Availability

The original contributions presented in the study are included in the article/Supplementary material, further inquiries can be directed to the corresponding author.

## Footnotes

1
https://www.wjx.cn/
