# Supplementary material for: A mediation analysis of family members’ knowledge, attitudes, and practices in nutritional and dietary management for gastric cancer patients
Source: Front Med (Lausanne). 2026 Jan 9;12:1680862. doi: 10.3389/fmed.2025.1680862 (PMC12827519; doi:10.3389/fmed.2025.1680862)
Supplement: Supplementary file 1 [file Supplementary_file_1.docx]

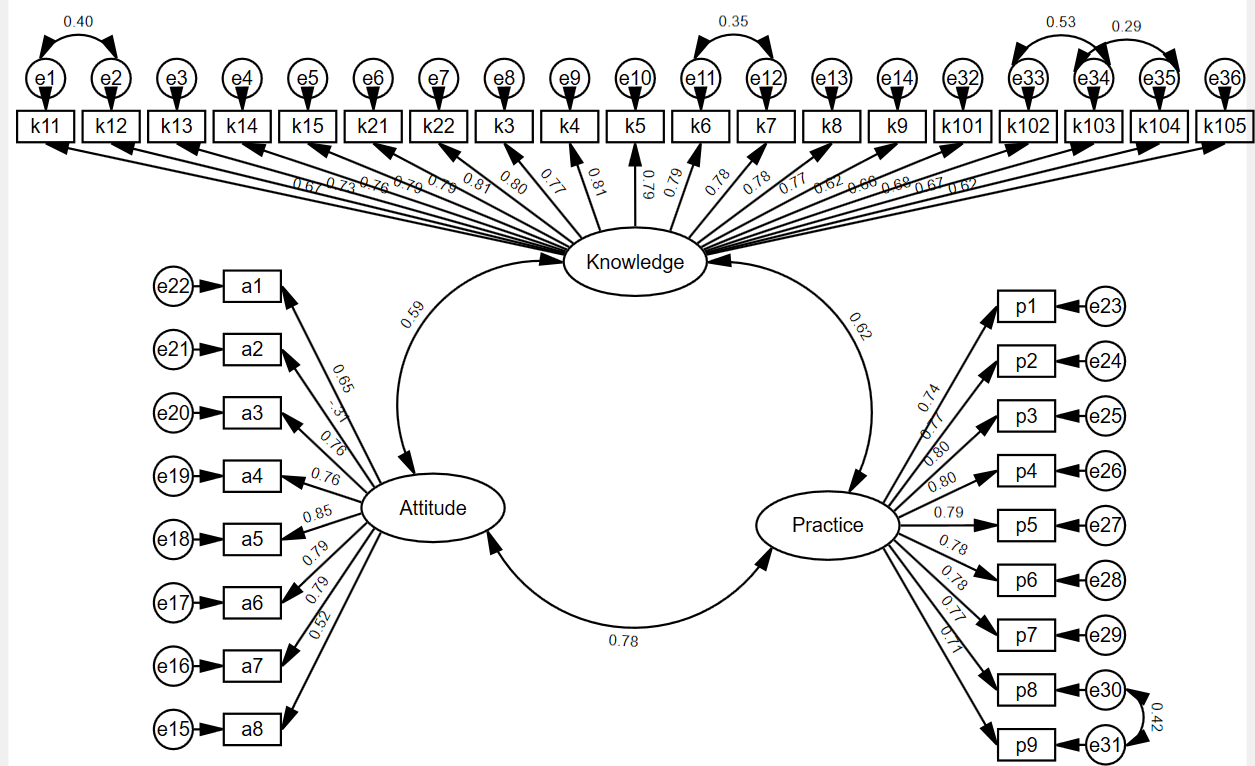


**Figure S1. CFA path**

**Table S1. CFA results**

|  | **ref** | **Measured results** |
| --- | --- | --- |
| **CMIN/DF** | 1-3 excellent, 3-5 good | 3.541 |
| **RMSEA** | <0.08 good | 0.079 |
| **IFI** | >0.8 good | 0.867 |
| **TLI** | >0.8 good | 0.856 |
| **CFI** | >0.8 good | 0.866 |
