# Supplementary material for: A mediation analysis of family members’ knowledge, attitudes, and practices in nutritional and dietary management for gastric cancer patients
Source: Front Med (Lausanne). 2026 Jan 9;12:1680862. doi: 10.3389/fmed.2025.1680862 (PMC12827519; doi:10.3389/fmed.2025.1680862)
Supplement: Supplementary file 2 [file Supplementary_file_2.doc]

| Questionnaire number: |
| --- |
| Dear Participant:  We are researchers from xxx Hospital. We sincerely invite you to participate in our research project. This study aims to understand the knowledge, attitudes, and practices of family members regarding nutritional care for gastric cancer patients, to serve as the basis for developing scientific intervention strategies, and may help many others in the future to improve their health conditions. Your participation in this study is voluntary, and the research has been approved by the Ethics Review Committee. If you agree to participate, please read the following instructions:   1. Please complete the questionnaire, the answer is not right or wrong, you only need to fill in according to the actual situation. You can ask us any questions in the process of answering, and please submit it in time after you finish it. 2. This study is a simple questionnaire survey, which will not harm your physical and psychological condition, but it will involve some privacy issues, such as your gender, age, etc. We will keep strict confidentiality and will not disclose your information, so please feel free to fill it out. 3. As a participant, you can keep yourself informed of the information related to this study and the progress of the study. If you decide to withdraw from the study, please let us know that your data will not be included in the results of the study.   Finally, we sincerely thank you for taking time out of your busy schedule to support our scientific research!  □I have been informed and agreed to the use of the collected data for scientific research.  Informed Consent Signature：  Date of participation：YYYY MM DD |

| **Part I Basic Information** | |
| --- | --- |
| **1.Your relationship with the patient is:** | a.Parents  b.Spouse  c.Children |
| **2.Your age: years.** | |
| **3.Your gender:** | a.Male  b.Female |
| **4.Your education:** | a.Primary and below  b.Junior high school  c.High school/secondary school  d.College/Bachelor's Degree  e.Master's Degree and above |
| **5. Your ethnicity：** | a.Han Chinese  b.Ethnic minority |
| **6. Your work status:** | a.Employed  b.Unemployed  c.Retired  d.Self-employed  f. Other |
| **7.In the past year, your family's monthly per capita income was (including in-kind income and rental income, etc.):______Yuan** | a.<2000  b.2000-5000  c.5000-10000  d.10000-20000  e.>20000  f.Reluctant to disclose |
| **8.Your marital status:** | a.Unmarried  b.Married  c.Divorced  d.Widowed |
| **9.Do you have child:** | a.Yes  b.No |
| **10.Do you have a smoking habit?** | a.Never smoked  b.Used to smoke  c.Still smoke |
| **11.Do you have a drinking habit?** | a.Never drank alcohol  b.Used to drink alcohol  c.Still drink alcohol |
| **12.Whether the patient has medical or other commercial insurance:** | a.Yes  b.No |
| **13.How long has the patient been diagnosed with gastric cancer (duration of disease)?** | a.Within half a year  b.Half a year to 1 year  c.1 to 2 years  d.More than 2 years |
| **14.Does the patient receive nutritional care for gastric cancer?** | a.Yes  b.No  c.Uncertain |
| **15.Have you participated in education or training on cancer nutrition care?** | a.Yes  b.No |
| **16.Do you have any underlying diseases or chronic illnesses?** | a.Hypertension  b.Hyperlipidemia  c.Diabetes  d.Tumor  e.Hepatitis  f.Asthma/Chronic obstructive pulmonary disease (COPD)  e.Other (e.g., respiratory, digestive system diseases)  f.No |
| **17.Stage of gastric cancer in the patient:** | a.Stage Ⅰ-Ⅳ  b.Stages I-II are early-stage gastric cancer  c.Stages III-IV are advanced stages  d.Uncertain |

| **Part II Knowledge of nutritional care for gastric cancer patients** | | | |
| --- | --- | --- | --- |
| **1. The main causes of malnutrition in gastric cancer patients are:** |  |  |  |
| **1.1) Anorexia and depression caused by the disease itself lead to reduced food intake. Among all tumors, gastric cancer has the highest incidence of anorexia and early satiety.** | a.Very familiar | b.Heard of | c.Unclear |
| **1.2) Difficulty in intake caused by mechanical factors.** | a.Very familiar | b.Heard of | c.Unclear |
| **1.3) Absorption and digestive disorders caused by the toxicity of chemotherapy drugs.** | a.Very familiar | b.Heard of | c.Unclear |
| **1.4) Factors combined with increased catabolism, such as infection or surgical treatment. Gastric cancer patients who also have smoking and drinking habits are prone to local infections when their granulocyte count drops during concurrent radiotherapy and chemotherapy.** | a.Very familiar | b.Heard of | c.Unclear |
| **1.5) Specific effects of gastric surgery: Among all gastrointestinal surgeries, gastric surgery has the most complications, the greatest impact on nutrition and metabolism, and the longest duration. Metabolic changes and absorption disorders caused by gastric resection and diversion should be given due attention, such as absorption disorders and deficiencies of iron, calcium, vitamin A, vitamin B12, and vitamin D caused by gastric juice loss, and digestion and absorption disorders of fats, proteins, and carbohydrates.** | a.Very familiar | b.Heard of | c.Unclear |
| **2. Negative effects of gastric cancer-associated malnutrition:** |  |  |  |
| **2.1) Weakening of the efficacy of radiotherapy and chemotherapy, increased risk of adverse drug reactions, and reduced skeletal muscle mass and function.** | a.Very familiar | b.Heard of | c.Unclear |
| **2.2) Increased chances of postoperative complications and nosocomial infections, prolonged hospital stay, increased incidence of complications and mortality, deterioration of patients' quality of life, and increased medical expenses.** | a.Very familiar | b.Heard of | c.Unclear |
| **3.** **The approaches to nutritional therapy for gastric cancer patients include enteral nutrition (oral, tube feeding) and parenteral nutrition (intravenous).** | a.Very familiar | b.Heard of | c.Unclear |
| **4.** **After gastric cancer surgery, the stomach's volume significantly decreases, and overeating can lead to symptoms such as bloating and nausea. Therefore, consuming smaller, more frequent meals can alleviate bloating symptoms.** | a.Very familiar | b.Heard of | c.Unclear |
| **5. After gastric cancer surgery, the stomach's grinding function is partially or completely lost, so the chewing function of the teeth needs to partially substitute for the stomach's role. Patients should eat slowly after surgery to prevent rapid food expulsion, which affects digestion and absorption, and to prevent dumping syndrome. Patients should rest in a semi-recumbent position after meals to prolong the emptying time of food for complete digestion and absorption.** | a.Very familiar | b.Heard of | c.Unclear |
| **6. During gastric cancer surgery, cutting the vagus nerve or pylorus removal can accelerate postoperative gastric emptying, increase gastric juice loss, and cause pancreatic and biliary dysfunction, leading to digestion and absorption disorders of fats, proteins, and carbohydrates. Increased excretion of carbohydrates, fats, and proteins in feces after surgery can lead to postoperative malnutrition in patients. Therefore, high-calorie, high-protein diets should be supplemented.** | a.Very familiar | b.Heard of | c.Unclear |
| **7. The highest level of nutrition is balance, and special attention should be paid to deficiencies in vitamins and trace elements after gastric cancer surgery. Patients can consume moderate amounts of liver, red meat, seafood, milk, various soy products, and dairy products.** | a.Very familiar | b.Heard of | c.Unclear |
| **8. Patients can engage in appropriate exercises when their bodies have not fully recovered after gastric cancer surgery, such as walking, jogging, Tai Chi, and aerobics, to activate muscles, enhance physical strength, promote gastrointestinal motility, help digest food, improve appetite, and increase food intake.** | a.Very familiar | b.Heard of | c.Unclear |
| **9. Prolonged negative emotions such as loneliness, sadness, and despair can cause neuroendocrine disorders, weaken immune surveillance function, and lead to sudden proliferation of cancer cells. Therefore, maintaining a positive attitude is crucial for gastric cancer patients. When patients feel low or in poor mental condition, they should talk to family or friends, build confidence in overcoming the disease, and regulate their emotions through reading, watching TV, participating in recreational activities, and listening to music.** | a.Very familiar | b.Heard of | c.Unclear |
| **10. Follow medical advice and use medication appropriately:** |  |  |  |
| **10.1) Medication should be taken at fixed times and in accordance with medical advice.** | a.Very familiar | b.Heard of | c.Unclear |
| **10.2) Tumor drugs should not be arbitrarily reduced in dosage.** | a.Very familiar | b.Heard of | c.Unclear |
| **10.3) During treatment, adverse drug reactions should be closely monitored.** | a.Very familiar | b.Heard of | c.Unclear |
| **10.4) Drugs should be stored separately, and antitumor drugs should generally be stored in a light-resistant, dry place away from heat sources, with a temperature below 25 degrees Celsius being suitable. If there are children at home, special attention should be paid to keeping the drugs out of reach.** | a.Very familiar | b.Heard of | c.Unclear |
| **10.5) When taking analgesics, follow the three principles of medication: timely oral administration, administration on schedule, and administration according to the stepwise principle.** | a.Very familiar | b.Heard of | c.Unclear |

| **Part III Attitude to nutritional care for gastric cancer patients** | | | | | |
| --- | --- | --- | --- | --- | --- |
| **1.** **I believe that nutritional care for gastric cancer patients is key to improving their quality of life. (P)** | a.Strongly agree | b.Agree | c.Neutral | d.Disagree | e.Strongly disagree |
| **2.** **I find it challenging to provide nutritional care for gastric cancer patients, and I lack confidence and patience. (N)** | a.Strongly agree | b.Agree | c.Neutral | d.Disagree | e.Strongly disagree |
| **3.** **I believe that balanced nutrition for gastric cancer patients is one of the most important aspects of nutritional care that family members should pay attention to. (P)** | a.Strongly agree | b.Agree | c.Neutral | d.Disagree | e.Strongly disagree |
| **4.** **I believe that family members of gastric cancer patients need professional training in nutritional care. (P)** | a.Strongly agree | b.Agree | c.Neutral | d.Disagree | e.Strongly disagree |
| **5.** **To improve the effectiveness of nutritional care, I believe that family members need to pay attention to the patient's psychological and emotional well-being. (P)** | a.Strongly agree | b.Agree | c.Neutral | d.Disagree | e.Strongly disagree |
| **6.** **I believe that regular follow-ups help to adjust nutritional care strategies in a timely manner for greater benefits. (P)** | a.Strongly agree | b.Agree | c.Neutral | d.Disagree | e.Strongly disagree |
| **7.** **I believe that patients often need encouragement from healthcare professionals and family members to boost their confidence and patience in nutritional therapy. (P)** | a.Strongly agree | b.Agree | c.Neutral | d.Disagree | e.Strongly disagree |
| **8.** **I believe that medical institutions do not provide enough education on nutritional care for gastric cancer patients. (P)** | a.Strongly agree | b.Agree | c.Neutral | d.Disagree | e.Strongly disagree |

| **Part IV Practice on nutritional care for gastric cancer patients** | | | | | |
| --- | --- | --- | --- | --- | --- |
| **1.** **I will proactively learn about nutritional care for gastric cancer patients. (P)** | a.Always | b.Often | c.Sometimes | d.Rarely | e.Never |
| **2.** **I will strictly control the patient's diet to achieve balanced nutrition as much as possible. (P)** | a.Always | b.Often | c.Sometimes | d.Rarely | e.Never |
| **3.** **When encountering problems, I will seek professional medical advice proactively to adjust the nutritional care strategy in a timely manner. (P)** | a.Always | b.Often | c.Sometimes | d.Rarely | e.Never |
| **4.** **I will actively observe any adverse reactions during the patient's home treatment process. (P)** | a.Always | b.Often | c.Sometimes | d.Rarely | e.Never |
| **5.** **I will encourage and accompany the patient to engage in moderate exercise. (P)** | a.Always | b.Often | c.Sometimes | d.Rarely | e.Never |
| **6.** **I will always pay attention to the patient's psychological fluctuations and care for their emotions. (P)** | a.Always | b.Often | c.Sometimes | d.Rarely | e.Never |
| **7.** **I will schedule regular follow-up appointments with the patient. (P)** | a.Always | b.Often | c.Sometimes | d.Rarely | e.Never |
| **8.** **I will actively participate in the process of developing nutritional care plans for patients with doctors and dietitians. (P)** | a.Always | b.Often | c.Sometimes | d.Rarely | e.Never |
| **9.** **I will actively participate in any educational activities on nutritional care for gastric cancer patients organized by medical institutions. (P)** | a.Always | b.Often | c.Sometimes | d.Rarely | e.Never |

| **Thank you again for your participation in completing our questionnaire, the information provided by your answers will be very valuable to us in the future!**  **Thank you for filling out our questionnaire！**  We would be honored to hear from you if you have any comments or suggestions about this research.  Opinions and Suggestions: (optional)  To ensure that this questionnaire is effective and to promote the smooth implementation of future follow-ups, we would be grateful if you could leave your contact details!  Your phone number: (optional) |
| --- |
